# Lenalidomide before and after Autologous Hematopoietic Stem Cell Transplantation in Multiple Myeloma

**DOI:** 10.1155/2012/712613

**Published:** 2012-05-30

**Authors:** S. A. Tuchman, N. J. Chao, C. G. Gasparetto

**Affiliations:** ^1^Division of Medical Oncology and Hematological Malignancies Program, Duke University Medical Center, Durham, NC 27710, USA; ^2^Division of Cellular Therapy and Hematological Malignancies Program, Duke University Medical Center, Durham, NC 27710, USA

## Abstract

Although multiple myeloma remains incurable outside of allogeneic hematopoietic stem cell transplantation, novel agents made available only in the last few decades have nonetheless tremendously improved the landscape of myeloma treatment. Lenalidomide, of the immunomodulatory class of drugs, is one of those novel agents. In the non-transplant and relapsed/refractory settings, lenalidomide clearly benefits patients in terms of virtually all meaningful outcomes including overall survival. Data supporting the usage of lenalidomide as part of treatment approaches incorporating high-dose chemotherapy with autologous stem cell support (ASCT) are less mature as pertains to such long-term outcomes and toxicity, and lenalidomide is not currently approved by regulatory agencies for use in the context of ASCT in either the United States or Europe. That said, relatively preliminary efficacy data describing lenalidomide as a component of ASCT-based treatment approaches to MM are indeed promising, and consequently lenalidomide's role in ASCT-based treatment strategies is growing. In this review we summarize existing data that pertains to lenalidomide in the specific context of ASCT, and we share our thoughts on how our own group applies these data to approach this complex issue clinically.

## 1. Introduction

Multiple myeloma (MM) is a malignancy of plasma cells that strikes roughly 20,000 and kills 10,000 US Americans yearly [1]. Outside of allogeneic stem cell transplantation, MM remains incurable, albeit increasingly treatable, thanks to the advent of novel agents including those that are currently approved by the United States Food and Drug Administration (FDA) and European Medicines Agency (EMA)—bortezomib, thalidomide, and lenalidomide. 

Regarding the latter, the initial phase one study of lenalidomide (Revlimid), then known as CC-5013, first appeared in the scientific literature in 2002 and attention rapidly focused on CC-5013's activity even in multiply relapsed and refractory MM (RRMM) [2]. That study and others led to the later definitive phase three MM-009 and 010 trials, which showed overall survival benefits for RRMM patients on lenalidomide and dexamethasone in contrast to those on dexamethasone alone [3, 4]. FDA and EMA approval for lenalidomide followed, and lenalidomide and dexamethasone became established as a standard of care for RRMM. Subsequent clinical trials have further explored the role of lenalidomide as a part of treatment strategies for newly diagnosed MM (NDMM) that both include or exclude high-dose therapy with autologous hematopoietic stem cell transplantation (ASCT), although lenalidomide remains without approval for usage in the setting of ASCT by either FDA or EMA. In the current discussion, we focus on lenalidomide specifically as part of ASCT-based therapy approaches for MM.

## 2. Lenalidomide Induction prior to ASCT

The combination of dexamethasone and thalidomide compared favorably, in a retrospective study, with the earlier induction standard of vincristine, doxorubicin (Adriamycin), and dexamethasone (VAD), and thalidomide plus dexamethasone became an attractive therapeutic option for NDMM. However, significant nonhematological toxicity affected a large proportion of patients [5]. Thalidomide's efficacy and toxicity both engendered interest in lenalidomide as a possibly more potent and less toxic analog of thalidomide that could replace thalidomide in both the ASCT and non-ASCT settings.

Lenalidomide's activity in NDMM pre-ASCT was unmistakable from the outset. In its first major, phase 2 study, namely, that of the RD regimen (lenalidomide and high-dose dexamethasone; see Table 1 for details regarding regimens), 91% of patients responded and 44% proceeded to ASCT after four cycles. Toxicity was excessive, and the toxicity profile in general resembled that seen in prior trials of high-dose dexamethasone alone. The follow-up randomized trial by the Southwest Oncology Group (SWOG) of three cycles of dexamethasone alone versus RD induction, followed in each arm by the same drugs given at lower doses as maintenance, confirmed lenalidomide's activity in NDMM, manifesting as significantly increased response rates. However, that trial also provided clear evidence of lenalidomide's toxicity when used with high-dose dexamethasone, for example, a 23.5% rate of venous thromboembolic events for RD despite aspirin prophylaxis versus 5% for dexamethasone alone (*P* < 0.001) [6]. These observations of high activity and toxicity early on in the SWOG study gave rise to subsequent study of lenalidomide with lower dose, that is, weekly, dexamethasone, largely as a result of requests by patient advocacy groups [7, 8]. 

**Figure 1:**
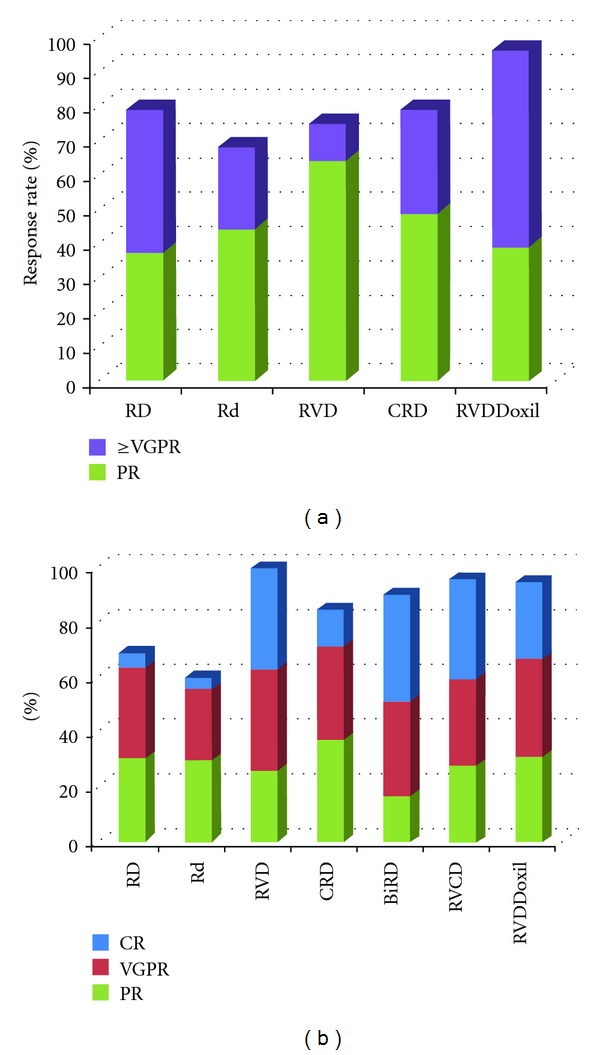
Reported response rates for lenalidomide-based induction regimens for MM. Rates depicted are those that could be ascertained either directly using reported data or as calculated using reported data. (a) Response rates after four cycles of therapy. Deeper response rates are not displayed due to inconsistent reporting in referenced sources. (b) Best response reached on study. Rates after four cycles could be envisioned as a measure of expected response pre-ASCT, whereas best response rate may represent a regimen's maximum potential, but only after more cycles than a patient would usually be administered as pre-ASCT induction. Data was gleaned from the following sources: RD and Rd [9]; RVD [11]; CRD [12]; BiRD [10]; and RVDDoxil [14].

The ECOG's E4A03 study (*n* = 445) included both ASCT candidates and noncandidates and was designed with the primary endpoint of testing noninferiority of lenalidomide given with low-dose (weekly) dexamethasone (i.e., the Rd regimen; Table 1) to RD. Patients on RD demonstrated more objective responses than patients taking Rd (overall response rate ORR 81% versus 70%, *P* = 0.008; Figure 1), but at the price of inferior one-year overall survival (87% one-year overall survival OS for RD versus 96% for Rd, *P* = 0.0002). Closer inspection reveals that the increased mortality with RD was likely associated with the higher rate of grade 3 or greater venous thromboembolic events, infection, or cardiac complications than Rd and that toxicity occurred primarily in the first four months of therapy. In terms of ASCT, 39.5% of patients in this study attempted ASCT after four cycles of induction, 98% of whom did so successfully. Among ASCT patients, median three-year OS was 92% and similar between the RD and Rd groups. RD and Rd both emerged as clearly effective regimens for pre-ASCT induction. Although the OS one-year benefit to Rd has resulted in the more widespread usage of low-dose dexamethasone than high-dose, for patients going to ASCT, one should recall that the survival benefit with Rd was specifically in patients not going for ASCT [9]. 

Since initial reports on E4A03, investigators have sought to build on the lenalidomide/dexamethasone backbone to create even more efficacious pre-ASCT regimens. Several have been described, and the result comprises a significant contribution to the increasingly complex combinations that constitute modern oncology; BiRD, RVD, CRD, RVCD, and RVDDoxil are perhaps the most robustly described examples. An overview discussion of each of these regimens follows. The reader will note the paucity of head-to-head studies of most of these regimens, and this discussion hence largely limits itself to comparisons of single-arm trials. The important caveats of cross-trial comparisons therefore apply: selection bias (i.e., differences in patient selection both for trial participation and for later ASCT), variable durations of planned duration of protocol therapy and followup, and reporting of different, often surrogate endpoints, among other limitations. We offer Figure 1 partially to visually summarize available data, but also to underscore the difficulty, if not impossibility, of selecting the “correct” induction regimen based on what we know about these combinations.

Starting with BiRD, Niesvizky et al. sought to improve upon their earlier experience with the combination of thalidomide, dexamethasone, and the macrolide antibiotic clarithromycin (Biaxin), the latter of which had preclinical data supporting both independent cytotoxicity and potentiation of dexamethasone's cytotoxic effect in MM [16]. Building on Rd, this group devised BiRD—Rd plus twice daily clarithromycin (Table 1). In a single-arm trial (*n* = 72), 90.3% of patients had an objective response with 73.6% of patients achieving a very good partial response (VGPR) or better (Figure 1). 25% of patients underwent ASCT after four or more cycles with a 5.5% (one patient) mortality rate. Two-year event-free survival for the ASCT group was 85.2% [10]. A separate case-control study comparing this cohort to matched patients who received Rd showed that BiRD was associated with notably deeper responses with induction (e.g., 73.6% versus 33.3% VGPR or better, *P* < 0.001) and a statistically insignificant trend toward improved OS. Grade 3 or greater toxicity was also increased with BiRD and was largely hematological. Important differences in these trials are worth noting, including that the median duration of therapy for BiRD was longer than Rd (11.8 versus 6 months) and BiRD patients undergoing ASCT did so much later (13.5 versus 5.9 months). The authors state that it was unclear to them why BiRD patients remained on therapy so much longer than Rd patients, but it stands to reason that the longer duration of therapy augmented both toxicity and ORR [17].

RVD (lenalidomide, bortezomib, and dexamethasone; Table 1)—a logical extrapolation of Rd to incorporate the proteasome inhibitor bortezomib—has perhaps gained the widest implementation by community oncologists after the initial phase 1/2 study demonstrated a 100% ORR and 74% VGPR or better rate in 35 phase 2 patients receiving a median of 10 cycles at the maximum tolerated dose (MTD) established in the earlier, phase 1 component of the study (Figure 1). 28 (42%) of all patients on protocol underwent ASCT at some point after cycle four with no significant difficulties reported. Among ASCT patients, the authors observed a 100% ORR with 57% of patients attaining VGPR or better. The median PFS for all patients at 18 months was 75%, and median OS had not been reached at the time of publication [11].

With CRD (cyclophosphamide, lenalidomide, dexamethasone; Table 1), investigators again sought to improve lenalidomide/dexamethasone, this time by adding weekly oral cyclophosphamide in three weeks of a four week cycle. In this single arm trial (*n* = 53), the ORR was 85% with 47% of patients achieving VGPR or better (Figure 1). 58% of patients at some point went on to attempt ASCT, but, in 25% of cases, hematopoietic stem cell mobilization with G-CSF was unsuccessful. The majority of those patients could be salvaged using either cyclophosphamide or plerixafor. In those patients that actually underwent ASCT, no unexpected complications were noted, and, in all patients, the median duration of response was 30.9 months. In general, the regimen was well tolerated, with the main toxicity being neutropenia; almost 60% of patients experienced grade 3 or 4 neutropenia [12]. 

RVCD has also been tested, with the idea of combining lenalidomide, bortezomib, cyclophosphamide, and dexamethasone into a single pre-ASCT induction regimen. In the initial phase 1 EVOLUTION study—the single randomized study available for all the induction regimens under discussion—patients received RVD (*n* = 42), RVCD (*n* = 41), or VCD (bortezomib, cyclophosphamide, and dexamethasone; *n* = 32) (Table 1). An update for the subsequent phase 2 component of this trial was presented at the American Society of Hematology meeting in 2010, in which additional 17 patients were given modified VCD (mVCD), in which the week three treatment break for cyclophosphamide was eliminated. Ultimately all regimens proved to have significant activity as measured by ORR, ranging from 78% (VCD) to 100% (mVCD) and ≥VGPR rates ranging from 41% (VCD) to 90% (RVCD and mVCD; Figure 1). Toxicity was similar and manageable across all groups. At the time of the first paper's publication, 13 of the original 25 patients had undergone ASCT with only one patient requiring a second attempt at stem cell collection. Specific details regarding mobilization are not yet reported, nor is longer-term followup available for this trial [13, 18].

Lastly, RVDDoxil—that is, RVD with liposomal doxorubicin (Doxil; Table 1)—has also been examined in the context of pre-ASCT induction for NDMM. In the published phase 1/2 study (*n* = 72 evaluable patients), 39 patients were treated at what was found to be the MTD. 58 patients (81%) underwent stem cell collection after a median of 3 to 8 cycles, 40 of whom (69%) received cyclophosphamide, plerixafor, or both in addition to standard G-CSF for stem cell mobilization. 49 patients (68%) proceeded to ASCT after four to eight cycles of RVDDoxil. ORR in all patients (ASCT and non-ASCT) receiving the MTD was 95% with 64% achieving VGPR or better at any point (Figure 1). ASCT proceeded without unexpected complications in all patients. Long-term followup is unavailable, but 18-month PFS for all patients was 81.6%; 93.5% for patients who underwent ASCT and 64.3% for patients who did not. Similar to the other studies discussed, hematological toxicity, neuropathy, fatigue were the primary manifestations of toxicity, although they were generally manageable with appropriate dose-reductions [14].

With the exception of perhaps EVOLUTION, these clinical trials will likely not greatly aid clinicians in sorting out the obvious question of which induction regimen is best for the patient moving toward ASCT. Future comparative studies with long-term followup of meaningful endpoints are critical, particularly as the picture becomes even more complex with upcoming trials looking at combinations of the latest generation of novel agents, such as carfilzomib and pomalidomide. Only the earliest data exist as of yet for those agents in the pre-ASCT setting, but those data suggest that these agents too can induce very deep responses pre-ASCT. Jakubowiak et al., for example, reported their pilot study in an oral abstract detailing carfilzomib, lenalidomide, and dexamethasone (CarRD—our abbreviation; Table 1) as induction therapy for NDMM. CarRD preliminarily appears to be at least as potent as the established regimens with published data, with 65% of patients reaching VGPR or better [15].

### 2.1. Stem Cell Mobilization and Collection after Lenalidomide-Based Induction

Stem cell mobilization into the peripheral blood and subsequent stem cell collection is the critical prelude to ASCT, with the usual aim of collecting enough cells to perform two ASCTs. Given that one of lenalidomide's most common toxicities is myelosuppression, from early on investigators have considered whether lenalidomide could damage hematopoietic stem cells and hinder G-CSF-induced mobilization. Further studies have examined whether cyclophosphamide or plerixafor could be used to overcome difficulties in mobilization that may be linked to lenalidomide-based induction.

Kumar et al. retrospectively reviewed 376 eligible patients who had undergone stem cell collection within 12 months of starting MMf therapy. 12.8% of patients had received lenalidomide and dexamethasone-based induction, whereas the others received VAD, thalidomide + dexamethasone, or dexamethasone alone. For mobilization, 64.3% of all patients received G-CSF alone and 33.6% received G-CSF with cyclophosphamide. The decision to employ the latter was made based on whether patients appeared to have “active disease,” defined as the presence of circulating plasma cells at the time of mobilization. Of patients receiving G-CSF alone, three completely failed to mobilize—all had received lenalidomide for greater than six months. Furthermore, day one collection yield and total daily collection of stem cells correlated inversely with duration of lenalidomide therapy. Among patients who received cyclophosphamide-based mobilization, only five previously took lenalidomide as part of their induction. Despite impaired mobilization, however, no difference in engraftment kinetics was evident (denoting length of time until peripheral blood cell count recovery after reinfusion of stem cells) [19]. Other retrospective studies have since confirmed the link between lenalidomide and impaired mobilization. That said, the duration dependency has not been evident in all studies, meaning that a longer duration of lenalidomide therapy in some studies has not predicted greater difficulty with mobilization [20, 21]. Given the episodic difficulty of G-CSF mobilization after lenalidomide induction, subsequent studies have looked at cyclophosphamide and plerixafor as potential tools for overcoming difficulties with mobilization.

Cavallo et al. prospectively studied 346 patients who had received four cycles of Rd followed by G-CSF and cyclophosphamide for mobilization. In 21% of patients, adequate stem cells for two ASCTs could not be collected on the first attempt; they therefore went on to a second cyclophosphamide- and G-CSF-based mobilization. 8% of patients still had inadequate cells for even one ASCT after the second attempt and hence could not undergo ASCT. An additional 9% had enough cells for only one transplant, that is, 17% of patients had what would be considered a suboptimal collection using the gold standard mentioned. Engraftment kinetics were unimpaired. With 91% of patients achieving a successful mobilization at least for one ASCT, however, four cycles of Rd followed by mobilization with G-CSF and cyclophosphamide were felt by the authors to be a reasonable strategy for patients going for ASCT.

The C-X-C chemokine receptor type 4 (CXCR4) antagonist plerixafor may also mitigate lenalidomide-related impairment of stem cell mobilization. In one study, plerixafor was given with G-CSF as an initial attempt at mobilization (*n* = 20) or for remobilization in the case of an initial failed stem cell mobilization (*n* = 40) and results were retrospectively studied. Patients in both groups had received a median of roughly four cycles of lenalidomide-containing induction (range 1–20). 5% of patients receiving front-line plerixafor versus 52.5% of patients receiving it as a remobilization strategy failed to achieve the goal of collection for two ASCTs, although for most patients collection was adequate for at least a single ASCT. It appeared that patients undergoing remobilization who had received >3 cycles of lenalidomide induction had a greater incidence of mobilization failure despite plerixafor, although small sample sizes precluded drawing definitive conclusions. Engraftment kinetics were again acceptable. In summary, it appears that plerixafor can to some degree overcome lenalidomide-related impairment of stem cell mobilization, but not entirely [22].

### 2.2. Lenalidomide in the Pre-ASCT Setting: Our Approach

Our approach to lenalidomide in the induction setting for ASCT patients is as follows. Existing data support, albeit not definitively, the concept that deep remissions going into ASCT are a desirable aim—in many studies, they correlate with long-term survival. Clearly, deep remissions in an individual patient could reflect either disease biology OR therapy selection, and so a causal link between induction therapy selection and OS is currently lacking. We would refer the reader to astute discussions on this controversial topic that have been published already [23–25]. Caveats notwithstanding, we believe that the extremely high response rates seen with short-course, initial induction regimens such as those discussed above, taken in combination with early hints at unprecedented post-ASCT PFS durations and manageable toxicity, will eventually translate into improvements in OS as well. Furthermore, limiting the duration of therapy limits toxicity in general, including perhaps lenalidomide-mediated impairment of stem cell collection. For that reason, we believe in “hard and fast” induction, in which we most commonly offer triple-drug regimens to fit patients prior to ASCT—either RVD as noted above, or cyclophosphamide, bortezomib, and dexamethasone, depending on clinical circumstances. We usually do not utilize four-drug regimens, such as RVCD or RVDDoxil, because response rates do not seem to be markedly improved as compared to three-drug regimens (Figure 1), yet the potential for toxicity generally rises.

Other groups have reported on other pre-ASCT triplet regimens such as bortezomib, thalidomide, and dexamethasone [26]; bortezomib, doxorubicin, and dexamethasone [27]. Those are also valid and well-tested options, but a comprehensive discussion of all described pre-ASCT induction regimens goes beyond the scope of this lenalidomide-focused paper. Truly, with the plethora of currently available data including unfortunately very few randomized trials, many of these induction regimens could be argued for. Consequently selection of a regimen presently depends heavily on provider preference and patient comorbidities. Randomized clinical trials are clearly needed to sort through the existing equipoise, so the field can move beyond personal and institutional preferences into an era of evidence-based selection of induction regimens.

Whatever the specific regimen, we optimally limit duration of therapy to four but no more than six cycles of any lenalidomide-containing induction prior to stem cell collection. For patients who do receive lenalidomide with their induction, we generally mobilize stem cells with G-CSF and cyclophosphamide 4 g/m^2^, and we add plerixafor in cases of poor mobilization with the first two agents.

### 2.3. Lenalidomide Consolidation and Maintenance after ASCT

Early studies investigating the long-term usage of agents such as thalidomide and interferon-alpha were challenging, in the sense that interferon was overly toxic with minimal benefit [28] and thalidomide, although perhaps more efficacious, was also toxic and most patients could not tolerate it on the long term [29–31]. With the idea that lenalidomide may offer a more potent, less toxic maintenance therapy, several studies have examined the role of lenalidomide after ASCT. Followup of the two major trials driving the current discussion remains of relatively short duration, and the most recent data are only available in abstract form at the time at which we write this paper.

The CALGB 100104 trial has generated considerable excitement for lenalidomide maintenance. 568 patients who had received a variety of induction regimens usually including at least one novel agent and who had stable disease or better after single ASCT were randomized to either lenalidomide or placebo maintenance given at 5–15 mg daily, based on tolerance. An initial benefit of almost doubling of time to progression led to unblinding and cross-over to lenalidomide for 87% of placebo patients [32]. Data presented early in 2011 in an oral abstract supported an OS benefit based on an intention-to-treat analysis despite the crossover; 9% of lenalidomide patients died versus 16.1% of placebo patients with a median followup of 17.6 months (*P* < 0.019). Exploratory analyses suggest that the benefit of lenalidomide was present regardless of beta-2-microglobulin level but statistical interactions between the effect of lenalidomide and other risk factors, such as cytogenetic or fluorescent in situ hybridization (FISH) abnormalities, have not yet been reported [33].

Conversely, the Intergroupe Francophone du Myelome (IFM) 2005-02 lenalidomide maintenance trial has not confirmed the prolongation of OS despite longer median followup of 34 months. In this study, 614 patients who received either VAD +/− DCEP (dexamethasone, cyclophosphamide, etoposide, and cisplatin), or bortezomib and dexamethasone, as induction prior to ASCT were administered two cycles of lenalidomide consolidation, at 25 mg daily for three of four weeks. Subsequently patients were randomized to placebo or continuous lenalidomide 10–15 mg daily until relapse. The trial completed in mid-2010 with 34 months of median followup after randomization. Although lenalidomide almost doubled PFS (42 versus 24 months, hazard ratio HR 0.46, *P* < 10^−8^), definitive evidence for an OS benefit has not yet been reported. To our knowledge, at the time of writing this paper, subgroup analyses (i.e., examination of outcomes in patients with high-versus standard-risk MM) are not yet available [34].

Further data will be forthcoming from ongoing trials, such the Blood and Marrow Clinical Trials Network (CTN) 0702 protocol studying patients who have completed induction and who are then randomized to single ASCT, tandem ASCT, or single ASCT followed by four cycles of RVD consolidation. Additionally, a cooperative study between the Dana-Farber Cancer Institute and IFM is investigating shorter-course RVD pre- and post-ASCT versus longer RVD induction without ASCT as part of the initial treatment strategy. In all arms for both studies, patients will receive lenalidomide maintenance. These trials and others will help to clarify the role of lenalidomide for patients undergoing ASCT.

### 2.4. Lenalidomide Maintenance and Secondary Malignancies

Although maintenance lenalidomide is tolerable for patients, generally causes few symptoms, and carries likely clinical benefits as pertains to long-term outcomes, it may come at the price of secondary malignancies (SMs).

The above CALGB trial demonstrated more SMs in patients on lenalidomide maintenance: 7.7% of 231 patients on lenalidomide versus 1.7% of patients on placebo [33]. The IFM trial similarly showed SMs in 23 of 306 patients (7.5%) on lenalidomide versus 6 of 302 patients (2%) on placebo maintenance [34]. In both trials, SMs constituted a diverse collection of hematological and solid tumors. Further data from the IFM trial preliminarily hint at two key factors: (1) the increased incidence of SMs emerged most prominently 24 months after randomization; (2) in multivariate analysis, predictors of SMs included not only lenalidomide maintenance, but also advanced age, high ISS stage, male gender, and DCEP induction therapy. Cytogenetics did not predict SMs [34]. Current analyses are interrogating to what extent the inclusion of the more leukemogenic DCEP regimen on the IFM trial could explain at least part of these differences, but the continued controversy on this subject is highlighted by the fact that that placebo patients on the CALGB trial were offered cross-over into lenalidomide maintenance, whereas the IFM has stopped lenalidomide in that study, notably after patients had received 24 months of lenalidomide maintenance already. Further clues regarding the development of SMs may come from three other large trials of prolonged lenalidomide in non-ASCT candidates with MM, in which a very low incidence of SMs has been observed [35, 36]. As an example, Palumbo et al. reported their non-ASCT trial in which patients were randomized to melphalan and prednisone (MP); melphalan, prednisone and lenalidomide induction without maintenance (MPR); or MPR induction followed by lenalidomide maintenance (MPR-R). SMs occurred in 2 of 153, 6 of 152, and 4 of 150 patients on MP, MPR, and MPR-R, respectively. These rates were statistically equivalent [36]. Given these data showing virtually no increase in SMs in non-ASCT patients on lenalidomide long term, it has been hypothesized that the high-dose alkylator (i.e., melphalan) may play a key role in the development of post-ASCT SMs when lenalidomide maintenance is employed.

### 2.5. Lenalidomide Post-ASCT: Our Approach

Our group favors maintenance therapy after ASCT. The doubling of PFS in most trials with lenalidomide and the OS benefit in the CALGB trial weigh heavily in favor of that agent despite the small but real risk of SMs after ASCT. It is germane to the discussion of our practice to also mention that bortezomib too has growing evidence favoring its use in maintenance, especially in high-risk patients. When given at some point during ASCT-based therapy (in some trials only during induction, in others as maintenance), bortezomib mitigates, but does not eliminate entirely, the poor-prognosis implications of genetic markers such as the t(4; 14) chromosomal translocation [37], and, more recently, deletion of 17p in a trial by the HOVON cooperative group [38]. As a result of these emerging data, our general practice is to employ lenalidomide in the majority of MM patients after ASCT who have standard-risk cytogenetics and FISH, regardless of depth of response, and bortezomib in patients with high-risk markers such as 17p deletion. We do not prespecify a particular duration of maintenance with either agent, although data from ongoing maintenance trials may show in the future that limiting the time length of maintenance therapy may be beneficial.

## 3. Conclusions

This is an exciting time to care for MM patients. Novel agents such as lenalidomide and bortezomib have markedly lengthened survival for patients with MM and for the first time, we can begin to imagine turning MM into a chronic disease-like hypertension, diabetes, or chronic myelogenous leukemia. ASCT candidates especially enjoy a list of treatment options that continues to expand. Lenalidomide specifically is growing in importance in all stages of therapy for the ASCT patient, and rightfully so, given its capacity to induce deep remissions and extend both disease-free and overall survival without excess toxicity in most cases. How exactly to optimally incorporate lenalidomide into MM therapy is becoming clearer with time, but existing data can be difficult to interpret given the panoply of single-arm trials with relatively short followup and incomplete reporting of long-term, meaningful outcomes. Attention to long-term followup from large, randomized trials currently in progress will presumably enable us to employ this highly effective agent in a manner that achieves maximum benefit.

## Figures and Tables

**Table 1:** Select lenalidomide-based, pre-ASCT induction regimens for NDMM.

RD	Lenalidomide 25 mg orally days 1–21 and dexamethasone 40 mg orally days 1–4, 9–12, 17–20. 28 day cycles [9]
Rd	Lenalidomide 25 mg orally days 1–21 and dexamethasone 40 mg orally weekly. 28 day cycles [9]
BiRD	Clarithromycin 500 mg orally twice daily continuously, starting on day 2 of cycle 1; lenalidomide 25 mg orally days 3–21 of cycle 1, then days 1–21 of later cycles; dexamethasone 40 mg orally days 1, 2, 3, 8, 15, and 22 of cycle 1, then days 1, 8, 15, and 22 of later cycles. 28 day cycles [10]
RVD	Lenalidomide 25 mg orally days 1–14; bortezomib 1.3 mg/m^2^ IV days 1, 4, 8, 11; dexamethasone 20 mg orally days 1, 2, 4, 5, 8, 9, 11, 12. 21 day cycles [11]
CRD	Cyclophosphamide 300 mg (fixed dose) orally days 1, 8, and 15; lenalidomide 25 mg orally days 1–21; dexamethasone 40 mg orally days 1, 8, 15, 22. 28 day cycles [12]
RVCD	Lenalidomide 25 mg orally days 1–14; bortezomib 1.3 mg/m^2^ IV days 1, 4, 8, 11; cyclophosphamide 500 mg/m^2^ orally days 1 and 8; dexamethasone 40 mg orally days 1, 8, 15. 21 day cycles [13]
RVDDoxil	Lenalidomide 25 mg orally days 1–14; bortezomib 1.3 mg/m^2^ IV days 1, 4, 8, and 11; dexamethasone 20 mg orally days 1, 2, 4, 8, 11, and 12 for cycles 1–4 and 10 mg on the same schedule for later cycles; liposomal doxorubicin 30 mg/m^2^ IV on day 4. 21 day cycles [14]
CarRD	Carfilzomib 20–36 mg/m^2^ days 1, 2, 8, 9, 15, 16; lenalidomide 25 mg orally days 1–21; dexamethasone 20–40 mg days 1, 8, 15, and 22. 28 day cycles (dose of carfilzomib and dexamethasone was variable in this phase 1/2 study) [15]
